# Thermoresponsive Hyaluronate-Based Nanogels for Enhanced Phenanthriplatin Delivery in Cisplatin-Resistant Ovarian Cancer

**DOI:** 10.1021/acs.biomac.5c00692

**Published:** 2025-07-22

**Authors:** Filip Latečka, Tamara Juriňáková, Lukáš Münster, Monika Muchová, Michal Masařík, Anton Kuchynski, Petr Humpolíček, Michaela Fojtů, Jan Vícha

**Affiliations:** 1 Centre of Polymer Systems, Tomas Bata University in Zlín, tř. Tomáše Bati 5678, 760 01 Zlín, Czech Republic; 2 Department of Pathophysiology, Faculty of Medicine, Masaryk University, Kamenice 5, CZ-625 00 Brno, Czech Republic; 3 Department of Physiology, Faculty of Medicine, Masaryk University, Kamenice 5, CZ-625 00 Brno, Czech Republic; 4 BIOCEV, First Faculty of Medicine, Charles University, Průmyslová 595, 252 50 Vestec, Czech Republic; 5 Department of Fat, Surfactant and Cosmetics Technology, Faculty of Technology, Tomas Bata University in Zlín, nám. T. G. Masaryka 5555, 760 01 Zlín, Czech Republic

## Abstract

Stimuli-responsive hyaluronic acid carriers face limitations due to limited carboxyl groups, which are divided between drug conjugation and functional modifications. Thermoresponsive nanogels based on selectively oxidized hyaluronan (2,3-dicarboxy hyaluronate, DCH) grafted with poly­(*N*-isopropyl acrylamide) (pNIPAM) were developed for phenanthriplatin (PhPt) delivery. Sequential oxidation after pNIPAM grafting introduced additional carboxylic groups, enabling a more efficient drug loading and controlled release. Compared to nonoxidized pNIPAM-modified HA, this approach achieved 3 times higher loading efficacy and significantly slower drug release. Upon PhPt loading, DCH-pNIPAM conjugates self-assembled into nanogels, with the drug binding mode (ionic vs covalent) influencing particle rearrangement and drug release behavior. Covalently bound PhPt showed reduced release compared to nonthermoresponsive controls. *In vitro* studies on ovarian cancer cell lines, including cisplatin-resistant variants, demonstrated up to an 18-fold increase in cytotoxicity versus free PhPt. These nanogels offer a promising strategy for enhancing drug efficacy, reducing off-target effects, and overcoming resistance in cancer therapy.

## Graphical abstract

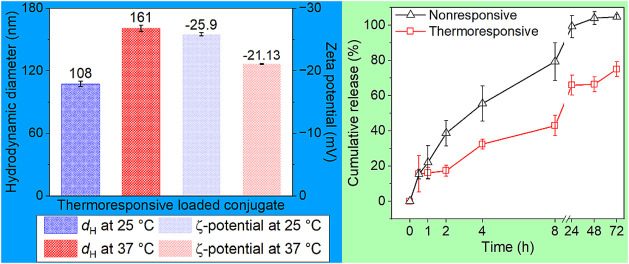

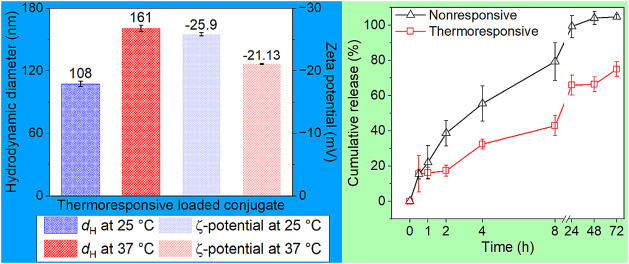

## 1 Introduction

Hyaluronic acid (HA) is a linear glycosaminoglycan formed of d-glucuronic acid (GA) and *N*-acetyl-d-glucosamine (NAG) units linked by alternating β-1,3 and β-1,4 glycosidic bonds. HA is found in the extracellular matrix (ECM), synovial fluid, connective tissues, and more.^1^ HA is also recognized by various cellular membrane receptors, such as CD44, CD168 (also known as RHAMM, receptor for hyaluronic acid mediated motility), hyaluronan receptors for endocytosis (HARE), lymphatic vessel endothelial hyaluronic acid receptors 1 (LYVE1), and Toll-like receptors (TLRs). The interactions involving these receptors play a crucial role in receptor-mediated hyaluronan internalization, angiogenesis, cell migration, proliferation, aggregation, and adhesion to ECM components.^2–6^ Given that many of these receptors are overexpressed in many malignant cell lines, HA has gathered significant interest among scientists developing anticancer drug delivery systems.^7,8^

Globally, ovarian cancer (OC) is the seventh most common malignancy among women and the eighth leading cause of cancer-related mortality.^9^ Nowadays, the standard treatment for ovarian cancer involves extensive tumor resection followed by postoperative platinum-based chemotherapy.^10^ The first and most well-known platinum-based chemotherapy drug cisplatin (CP) has been widely used for treating various cancers, including OC. The primary anticancer mechanism of CP involves forming intrastrand and interstrand DNA cross-links, causing DNA damage that arrests the cell cycle and induces cell death. Other mechanisms significantly contributing to cisplatin’s therapeutic effect include immunomodulation, disruption of tumor cell–microenvironment interactions, alteration of cancer cell mechanical properties, impact on cancer cell metabolism, and modulation of the intestinal microbiome.^11^ Although traditional platinum-based compounds such as CP remain the gold standard for ovarian cancer therapy, their use is often associated with the development of drug resistance, posing a significant challenge in oncology.^12^ Numerous mechanisms have been identified in the development of platinum-drug resistance, including the overexpression of drug efflux pumps, reducing intracellular drug concentration, increased DNA repair and damage, dysregulation of apoptosis, or the ability to avoid recognition by the host immune system.^13,14^ Various strategies to improve anticancer drug delivery and overcome drug resistance have thus been developed.

A number of these strategies involve HA and its derivatives,^15–18^ which are modified in various ways.^19–21^ Most of these methods focus on the carboxyl (−COOH) group of HA, which is a convenient target for advanced functional modifications as well as for direct binding of platinum­(II) complexes.^22^ Drugs thus compete with various other molecules for the same substrate. This can be particularly problematic for modern “smart” drug carriers, in which advanced modifications use the majority of the limited number of −COOH groups in HA.

This issue is addressed here by introducing an alternative approach for preparing thermoresponsive self-assembling nanogels based on a selectively oxidized hyaluronan (2,3-dicarboxy hyaluronate, DCH),^23,24^ grafted with thermoresponsive poly­(*N*-isopropyl acrylamide) (pNIPAM) for phenanthriplatin (PhPt) delivery. Initially, the pNIPAM is grafted to the −COOH group of HA (Figure 1), following earlier works of D’Este *et al.* ^25^ pNIPAM undergoes a sol–gel transition at a lower critical solution temperature (LCST) of approximately 32 °C, which is ideal for biological applications, and it is thus often used for the preparation of various biomaterials.^26–28^ The HA-pNIPAM conjugate prepared in this manner is thermoresponsive^25,29^ but suffers from the above-described limitations, as a significant part of the −COOH groups is used for pNIPAM substitution. Besides, bulky pNIPAM may sterically block the remaining free carboxyl groups from efficient drug binding. Therefore, sequential regioselective oxidation of HA-pNIPAM is performed, introducing additional carboxylic groups at C2 and C3 of d-glucuronic acid. This multiplies the number of available −COOH groups. Introduced groups are also at a distance and orientation ideal for the chelation of platinum-based drugs.^24^ Moreover, as these groups are located opposite to the pNIPAM chains conjugated at C6 d-glucuronic acid, they are not sterically hindered by pNIPAM. Combined, these factors will allow for more efficient drug binding and higher carrier capacity. This is a direct advantage compared to previously prepared HA-based thermoresponsive systems, in which drugs are simply adsorbed among HA chains or additional carrier molecules have to be introduced, leaving HA only in the role of targeting vector.^30–33^ In addition, this is one of the few examples in which selective oxidation is used after previous modifications have been used for the preparation of drug carriers. The only other example found in the literature uses TEMPO oxidation of cellulose nanofibrils for antibiotic delivery.^34^

**Figure 1:**
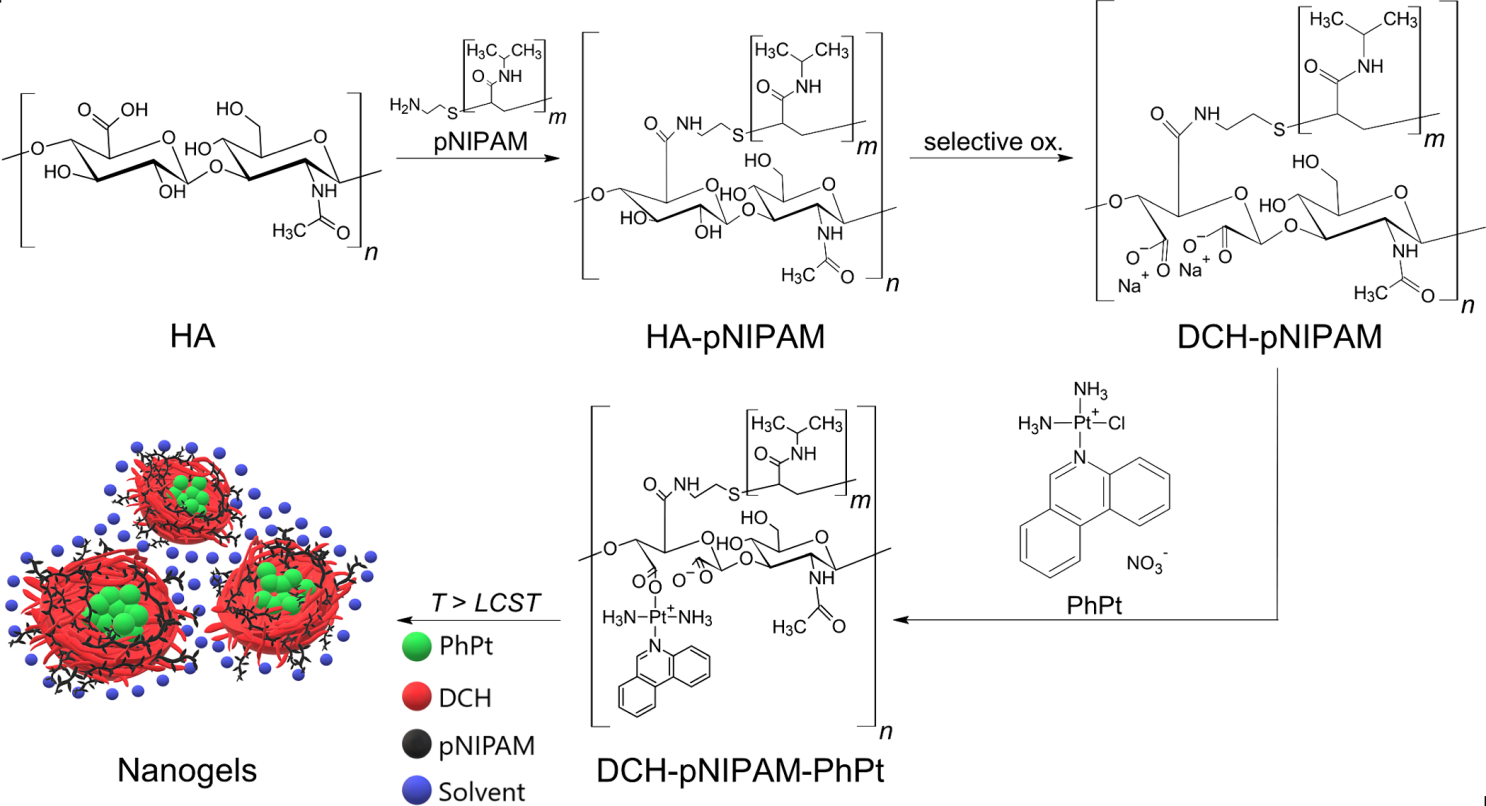
Schematic representation of the DCH-pNIPAM-PhPt nanogel preparation.

Loading of phenanthriplatin (PhPt), a next-generation platinum­(II) anticancer therapeutic,^35^ into the DCH-pNIPAM conjugates is straightforward due to their excellent solubility at laboratory temperature. Upon heating to body temperature (i.e., above the LCST), PhPt-loaded DCH-pNIPAM spontaneously self-assembles into nanogel particles, which protect the loaded drugs and reduce drug release. In contrast to CP, PhPt acts as a topoisomerase II poison by forming monofunctional DNA adducts that colocalize with topoisomerase II cleavage sites, stabilizing them.^36^ DNA topoisomerase II is a nuclear enzyme that plays a crucial role in the cell cycle by creating transient double-strand breaks in DNA. This allows for the manipulation of DNA topology, essential for processes such as DNA replication, transcription, and chromosome segregation.^37^ Different mechanisms of action, further assisted by advanced drug carriers, may help to overcome the CP resistance in OC cells.

Besides optimization of the synthesis, thorough characterization of PhPt-loaded DCH-pNIPAM nanogels and an *in vitro* evaluation of their biological activity in ovarian cancer cells, including their cisplatin-resistant variant, are performed in this work.

## 2 Methods

### 2.1 Materials

Sodium hyaluronate (HA, weight-average molecular weight *M*_w_ = 260 kDa and 2780 kDa, Contipro a.s., Czech Republic), amine terminated poly­(*N*-isopropyl acrylamide) (pNIPAM, *M*_n_ = 5.5 kDa, Aldrich chemistry), 4-(4,6-dimethoxy-1,3,5-triazine-2-yl)-4-methylmorpholinium chloride (DMTMM, 97%, Fisher Scientific), sodium periodate (NaIO_4_, Penta, Czech Republic), ethylene glycol (Penta, Czech Republic), sodium chlorite (NaClO_2_, 80%, Honeywell|Fluka), glacial acetic acid (CH_3_COOH, ≥99.8%, Sigma-Aldrich Co.), sodium acetate trihydrate (CH_3_COONa·3H_2_O, Penta, Czech Republic), hydrochloric acid (HCl, 35%, Penta, Czech Republic), sodium hydroxide (NaOH, ≥98%, Lachner, Czech Republic), potassium tetrachloridoplatinate­(II) (K_2_PtCl_4_, 99.9%, Alfa Aesar), phenanthridine (98%, Sigma-Aldrich), silver nitrate (AgNO_3_, ≥99.0%, Honeywell|Fluka), sodium chloride (NaCl, ACS, Supelco, Merck), sodium nitrate (NaNO_3_, 99.8%, Lachner, Czech Republic), disodium phosphate dodecahydrate (Na_2_HPO_4_·12H_2_O, 99.6%, VWR, Czech Republic), and phosphate-buffered saline pH 7.4 (PBS 7.4, Invitrogen, USA) were used. All chemicals were of analytical grade and were used without further purification. Ultrapure water (UPW, resistivity of 18.2 MΩ•cm) was used throughout the experiments.

### 2.2 Synthesis of DCH-pNIPAM

Synthesis of DCH-pNIPAM conjugates consists of four steps that are described in the following sections: the modification of HA by pNIPAM, oxidation of the HA-pNIPAM to dialdehyde derivative, the DAH-pNIPAM, its further oxidation to DCH-pNIPAM, and finally the loading of PhPt. Different reaction parameters, namely, the *M*_w_ of HA and the various weight ratios of HA:pNIPAM, were investigated. This is reflected in the coding of the final samples summarized in Table 1. Series 01 and 02 involve different molecular weights of the HA, while series A and B examine various reaction ratios between the HA and pNIPAM. To determine the impact of individual reaction steps on the *M*_w_ of HA, blank samples, which underwent all reaction steps without added pNIPAM, were prepared as well (samples C).

**Table 1:** Coding of the Samples and Their Properties

code	description
01	*M*_w_ of HA = 260 kDa
02	*M*_w_ of HA = 2780 kDa
A	HA:pNIPAM w/w 1:1
B	HA:pNIPAM w/w 1:2
C	without pNIPAM (blank)

#### 2.2.1 Synthesis of HA-pNIPAM

To prepare the HA-pNIPAM conjugates, the following general procedure was performed. Amine-terminated pNIPAM solution (25 mg/mL, UPW) was added to 500 mg of HA dissolved in UPW (25 mg/mL) to form a solution with a final concentration of HA 12.5 mg/mL (series A) and 8.3 mg/mL (series B). No pNIPAM was added to blank samples in series C, consisting of 100 mg of HA dissolved in UPW (20 mg/mL). The pH of the mixtures was adjusted to 6.5, and coupling agent DMTMM (*n*_HA_:*n*_DMTMM_ 1:1) was added. Reaction mixtures were gently stirred for 120 h at the laboratory temperature. The solutions containing HA-pNIPAM conjugates or HA (series C) were subsequently dialyzed using 14 kDa molecular weight cutoff (MWCO) dialysis tubing against 0.01 M NaCl solution for 48 h, followed by 24 h dialysis against UPW. The 14 kDa membrane was selected based on the difference between the molecular weight of pNIPAM (*M*_n_ = 5.5 kDa) and DCH (*M*_w_ between 43.0 kDa and 60.5 kDa; see below). It should allow the removal of pNIPAM and other low-molecular-weight compounds but not the carrier. The purified products were flash-frozen at −80 °C and lyophilized. Yields of all HA-pNIPAM (series A/B) and HA blank samples (series C) were nearly quantitative (over 90%).

#### 2.2.2 Synthesis of DAH-pNIPAM

NaIO_4_ (742.5 mg) was dissolved in 22.5 mL of UPW (33 mg/mL) and added dropwise to 22.5 mL of dissolved HA-pNIPAM conjugates containing 450 mg of HA (20 mg/mL, series A and B) to initiate the periodate oxidation (POX) of HA-pNIPAM. The blank sample (HA_C) was prepared similarly without pNIPAM addition. Reaction mixtures were stirred at 30 °C in a water bath for 24 h in the dark. The POX was terminated by adding excess ethylene glycol, and reaction mixtures were purified by dialysis (14 kDa MWCO dialysis tubing) against 0.01 M NaCl solution for 48 h, followed by 24 h of dialysis against UPW. Purified samples were filtered using 0.45 μm syringe filters, flash-frozen, and lyophilized. Yields of all DAH-pNIPAM (series A and B) and DAH (series C) were nearly quantitative (over 90%).

#### 2.2.3 Synthesis of DCH-pNIPAM

NaClO_2_ (724.8 mg) was added dropwise to DAH-pNIPAM conjugates dissolved in acetate buffer of pH 4.5 (0.045 M CH_3_COONa·3H_2_O and 0.055 M CH_3_COOH) containing 400 mg of DAH (series A and B) to initiate the secondary chlorite oxidation (SOX). The final concentration of NaClO_2_ was 0.1 M. The blank sample (DAH_C) was prepared similarly. Mixtures were stirred at laboratory temperature in the dark for 24 h. The SOX was terminated by adjusting the pH value to 8 with NaOH solution, and the products were purified by dialysis, as in section 2.2.2. Subsequently, the pH of dialyzed solutions was adjusted to pH 7.4 and filtered with 0.45 μm syringe filters, flash-frozen at −80 °C, and lyophilized. Yields of all DCH-pNIPAM (series A/B) and DCH (series C) were nearly quantitative (over 90%).

### 2.3 Synthesis of PhPt-Loaded DCH-pNIPAM Nanogels

*Cis*-diamminechlorido­(phenanthridine)­platinum­(II) nitrate, known also as phenanthriplatin (PhPt), was prepared according to the literature.^35^ To improve its binding with the conjugates, two different approaches to the “activation” step of PhPt were performed. Silver nitrate was added to PhPt solution (1 mg/mL) in the molar ratio of *n*_PhPt_:*n*_AgNO3_ 1:1, and the mixture was gently shaken at (a) 55 °C overnight and in the dark (designated as P1) or at (b) laboratory temperature for 72 h (designated as P2). The resulting [Pt­(NH_3_)_2_(H_2_O)­(phenanthridine)]­(NO_3_)_2_ mixtures were cooled, and the insoluble AgCl precipitate was removed by repeated filtration and centrifugation. In sample P1, 50 mL of the activated PhPt solution (1 mg/mL) was dropwise added to 100 mL of DCH-pNIPAM (0.5 mg/mL) and gently shaken at laboratory temperature in the dark for 48 h. In series P2, the pH of the PhPt solution was set to 7.0 before the addition to the DCH-pNIPAM solution, and the loading reaction was extended to 72 h. The loading procedure was otherwise identical. The weight ratio of PhPt:DCH-pNIPAM was 1:1 in both the P1 and P2 series. The products were purified by dialysis against UPW (MWCO = 3.5 kDa) at laboratory temperature for 4 h to remove unreacted PhPt. Purified products were flash-frozen in an ethanol bath at −80 °C and lyophilized.

### 2.4 Characterization of Conjugates

#### 2.4.1 Infrared Spectroscopy

Fourier transformed infrared spectroscopy (FT-IR) analysis was performed using a Nicolet 6700 FT-IR spectrometer (Thermo Fisher Scientific, USA) equipped with a diamond crystal in the ATR mode in a span of wavenumbers 4000–400 cm^–1^ (res. 4, scans 64), and the suppression of atmospheric gases was enabled.

#### 2.4.2 Gel Permeation Chromatography

Gel permeation chromatography (GPC) analysis was performed only on series C because thermoresponsive pNIPAM gelation could lead to column damage. All molecular weights measured by the GPC method in this work are apparent and are further addressed as weight-average molecular weight or molecular weight. A Waters HPLC Breeze chromatographic system (Waters, USA), equipped with a refractive index detector Waters 2414 (drift tube *T* = 60 °C) and a Tosoh TSK gel GMPW_XL_ column (300 mm × 7.8 mm × 13 μm, column *T* = 40 °C). The mobile phase consisted of a mixture of 0.1 M NaNO_3_ and 0.05 M Na_2_HPO_4_·12H_2_O. A flow rate of 0.8 mL/min was used for experiment measurements, and the data were processed with Empower Pro software. Pullulan polysaccharide calibration kit SAC-10 (10 pullulan standards with molecular range *M*_w_ = 342 g/mol to 805 000 g/mol, Agilent Technologies, USA) was used.

#### 2.4.3 Nuclear Magnetic Resonance Measurements

Proton nuclear magnetic resonance (^1^H NMR) spectra were recorded by using a JEOL 400 MHz NMR spectrometer (JEOL, Japan). The measurements were performed at *T* = 298 K in D_2_O with a sample concentration of 10 mg/mL. The degree of oxidation (DO) of the prepared DCH-pNIPAM samples was determined from a comparison of the intensity of the H5 signal of the oxidized units (4.0–4.2 ppm) and the H1′ signal of the unoxidized units, following earlier works.^23^ Grafting (*G*) was determined from a comparison of the Hα signal intensity of the pNIPAM unit (1.05 ppm) and the H5 signal of the DCH unit. The degree of substitution (DS) was estimated based on eq 1,

1 $$\mathrm{DS}=\frac{G}{\mathrm{DP}}$$

where the degree of polymerization (DP) was calculated by the following equation,

2 $$\mathrm{DP}=\frac{M_{n}}{M_{m}}$$

where *M*_n_ is the molecular weight of pNIPAM and *M*_m_ is the molecular weight of the pNIPAM monomer unit respecting the terminal amine (*M*_m_ = 115.778 g/mol).

#### 2.4.4 Transmission Electron Microscopy

Transmission electron microscopy (TEM) analysis of the PhPt-loaded DCH-pNIPAM and free DCH-pNIPAM conjugate was performed using a JEM-2100 transmission electron microscope (JEOL, Japan) operated at an acceleration voltage of 160 keV. The samples were dissolved in UPW (0.1 mg/mL) and sonicated (ice bath, 2 min, 40% intensity) using a homogenizer UZ Sonopuls HD 2070 kit (Bandelin, Germany). Subsequently, sample solutions were drop-cast on a 300-mesh copper grid coated with a Formvar membrane and gently dried at 25 °C (laboratory conditions) or at 40 °C in a dryer overnight.

#### 2.4.5 Dynamic Light Scattering Analysis

Zeta (ζ) potential and hydrodynamic diameter (*d*_H_) of the samples were determined using dynamic light scattering (DLS) on a Zetasizer ZETA NANO ZS ZEN3601 instrument (Malvern Instruments, U.K.) at 25 and 37 °C using DTS1070 cells and the Smoluchowski model. Samples were dissolved (1 mg/mL) in UPW or PBS 7.4 using the sonication process (ice bath, 2 min, 40% intensity). Subsequently, the samples were diluted to 0.05 mg/mL and measured.

#### 2.4.6 Analysis of Viscoelastic Properties

The viscoelastic behavior of the diluted samples was measured using a rotational rheometer Anton-Paar MCR 502 (Anton Paar, Austria) equipped with a cylinder geometry DG26.7-SN43705 (internal diameter 23.826 mm, external diameter 27.577 mm). Dissolved samples in UPW (20 mg/mL) were measured at span of temperature 25–40 °C. The measurement was performed at a constant strain of 1%, constant frequency of 0.1 Hz, and temperature gradient of 0.5 °C/min.

#### 2.4.7 Drug Release

Cumulative drug release was investigated using a setup mimicking *in vitro* conditions in which 10 mg of each conjugate was dissolved in 5 mL of PBS 7.4 and dialyzed (3.5 kDa MWCO dialysis tubing) against 95 mL of the same medium at 37 °C, while gently shaken.^38^ Aliquots of 5 mL from the dialysate were collected over 72 h and replaced with 5 mL of fresh media to conserve the volume. The released PhPt-aqua complex is in the following text also termed PhPt for the sake of clarity. The amount of released platinum was measured by the energy-dispersive X-ray fluorescence (XRF) spectrometer ARL Quant’X EDXRF Analyzer (Thermo Scientific, USA) using the calibration standards prepared by dissolving a known amount of PhPt in PBS 7.4.

### 2.5 Biological Evaluation

#### 2.5.1 Cell Culture

Two human carcinoma cell lines utilized in this study include A2780, a human ovarian cell line derived from the tumor tissue of an untreated patient, as well as its cisplatin-resistant variant, A2780/CP. A2780 cell line was cultivated in RPMI-1640 medium with 10% FBS, 1% antibiotics (penicillin 100 U/mL and streptomycin 0.1 mg/mL), and 0.7% HEPES. A2780/CP cell line was cultivated in the same medium, with cisplatin (1 μM) added during every second or third medium exchange. Cells were grown in an incubator at 37 °C in a humidified 5% CO_2_ mixture with ambient air.

#### 2.5.2 Evaluation of Cytotoxicity

Cancer cell lines were subjected to 02_DCH_A-P1, 02_DCH_A-P2, and PhPt treatments to study their biological activity. The cytotoxic potential of the nanogels was evaluated by an MTT assay. The MTT assay is a colorimetric assay based on the metabolic activity of mitochondrial oxidoreductases converting yellow MTT (tetrazolium salt (3-(4,5-dimethylthiazol-2-yl)-2,5-diphenyltetrazolium bromide) into formazan crystals. Cells were harvested and seeded into plates at concentrations of 6000–8000 cells per well in 200 μL of medium and incubated for 48 h. Afterward, the cell culture media were replaced by media solutions of 02_DCH_A-P1, 02_DCH_A-P2, and PhPt at concentrations ranging from 0 to 25 μM. After a 48 h incubation, the MTT solution was added, and the plate was incubated for an additional 4 h. Formazan crystals were dissolved in DMSO. The final absorbance of each well was measured at 570 nm by using a Cytation 3 Imaging multimode imaging reader. The data were analyzed by GraphPad Prism software, and IC_50_ concentrations were determined by dose–response nonlinear regression analysis. All measurements were performed in quadruplicate.

#### 2.5.3 Wound-Healing Assay

The wound-healing assay used for the evaluation of cell migration potential was monitored in real-time by the IncuCyte scratch wound migration system (IncuCyte S3, Sartorius). This system allows us to study the cell morphology and explore the differential biology of cell migration. Following cell passaging, each cell line was resuspended and seeded into Imagelock 96-well plates with an optimized number of cells per well in 100 μL of medium for each cell line. Once the cells reached 100% confluency, no-cell zones were made using the Incucyte 96-well Woundmaker Tool. Subsequently, after a gentle wash and medium change, the tested substances were added to the IC_25_ concentrations determined from the MTT tests. The data were processed and evaluated as changes in relative wound density over 48 h by Incucyte software.

## 3 Results and Discussion

### 3.1 Preparation of DCH-pNIPAM Conjugates

The composition of free pNIPAM, HA, DCH, and their pNIPAM-grafted conjugates was initially analyzed by FT-IR. The spectra of all oxidized samples show increased intensity of vibration at 1610 cm^–1^ (−COOH group) in comparison with the spectrum of source HA, as a result of HA’s successful oxidation to DCH (Figure 2a,b). Spectra of both series A and B samples also contain characteristic coupled valence vibrations of the amide group of pNIPAM at 3296 cm^–1^ (amide A), 1639 cm^–1^ (amide I), 1537 cm^–1^ (amide II), and the C–H bond vibration in the range of 2875–2970 cm^–1^, signifying a successful pNIPAM conjugation.

**Figure 2:**
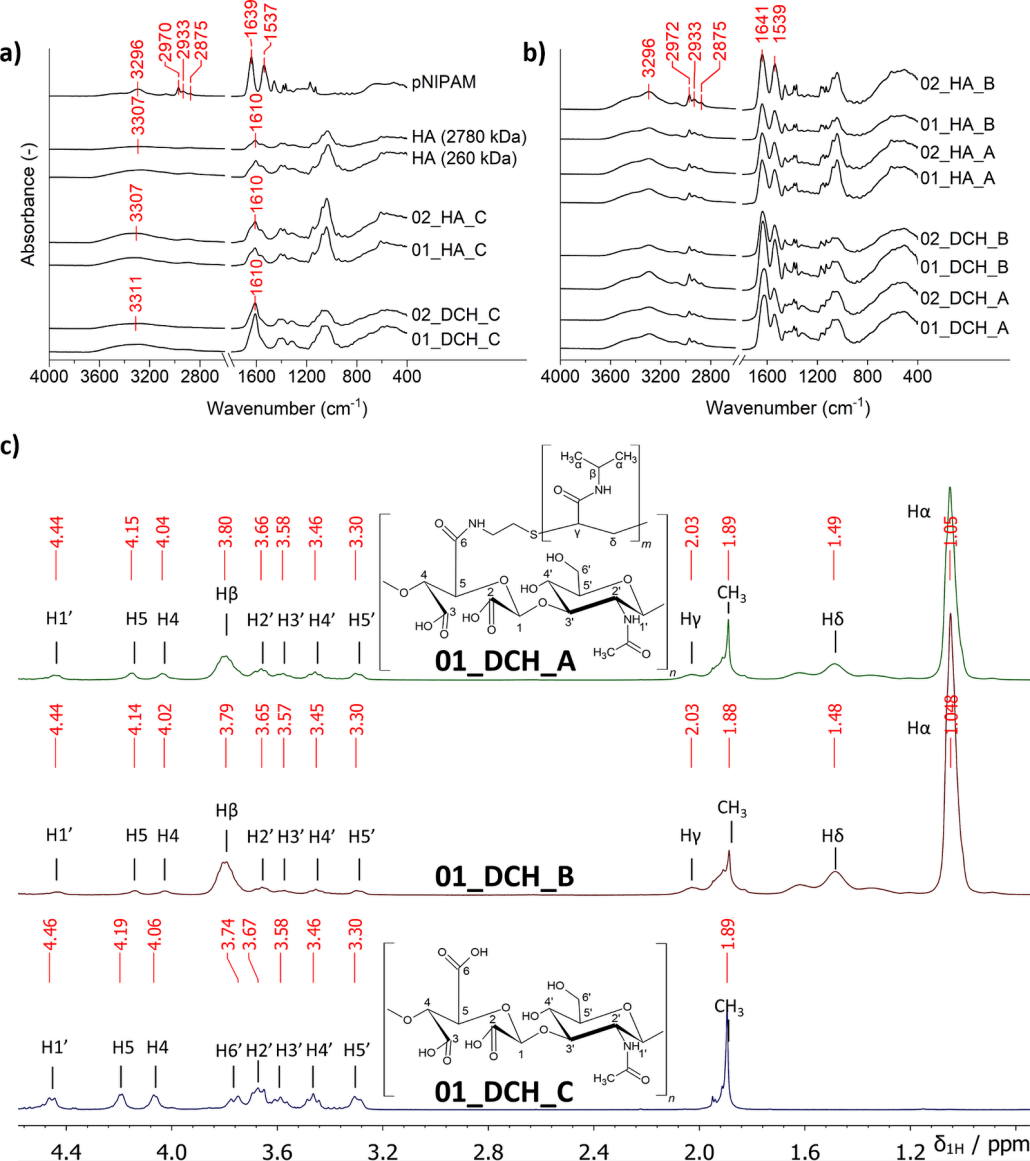
FT-IR spectra of (a) source HA, pNIPAM, and series C samples and (b) thermoresponsive conjugates from series A and B samples. (c) ^1^H NMR spectra of samples from series 01 (298 K, D_2_O).

Next, DCH and DCH-pNIPAM conjugates were analyzed by ^1^H NMR spectroscopy (Figure 2c). Only spectra of series 01 samples are shown, as those of series 02 were virtually identical. For a complete assignment of the DCH signals, see our previous work.^23^ NMR spectra confirmed that the oxidation of HA is limited to the GA unit. Signals of the NAG unit in 01_DCH_C are similar to native HA and are found mainly between 3.3–3.7 ppm, with the signal of H1’ deshielded to 4.44 ppm. The signal of H1 from the oxidized unit at 4.65 ppm is not shown in the spectra because of its overlap with the HDO signal. To exactly determine the DO (degree of oxidation), a comparison of integral intensities of oxidized (H5) and unoxidized groups (H1′) was performed; see section 2.4.2.1 for more details. All samples were found to be highly oxidized (DO ≥ 92%; see Figure 3a), as was expected due to the use of an optimized reaction procedure.^23^ The residual signal of the CH_3_ group of *N*-acetyl-d-glucosamine neighboring unoxidized d-glucuronic acid units is still observable at 1.93 ppm.^23^

**Figure 3:**
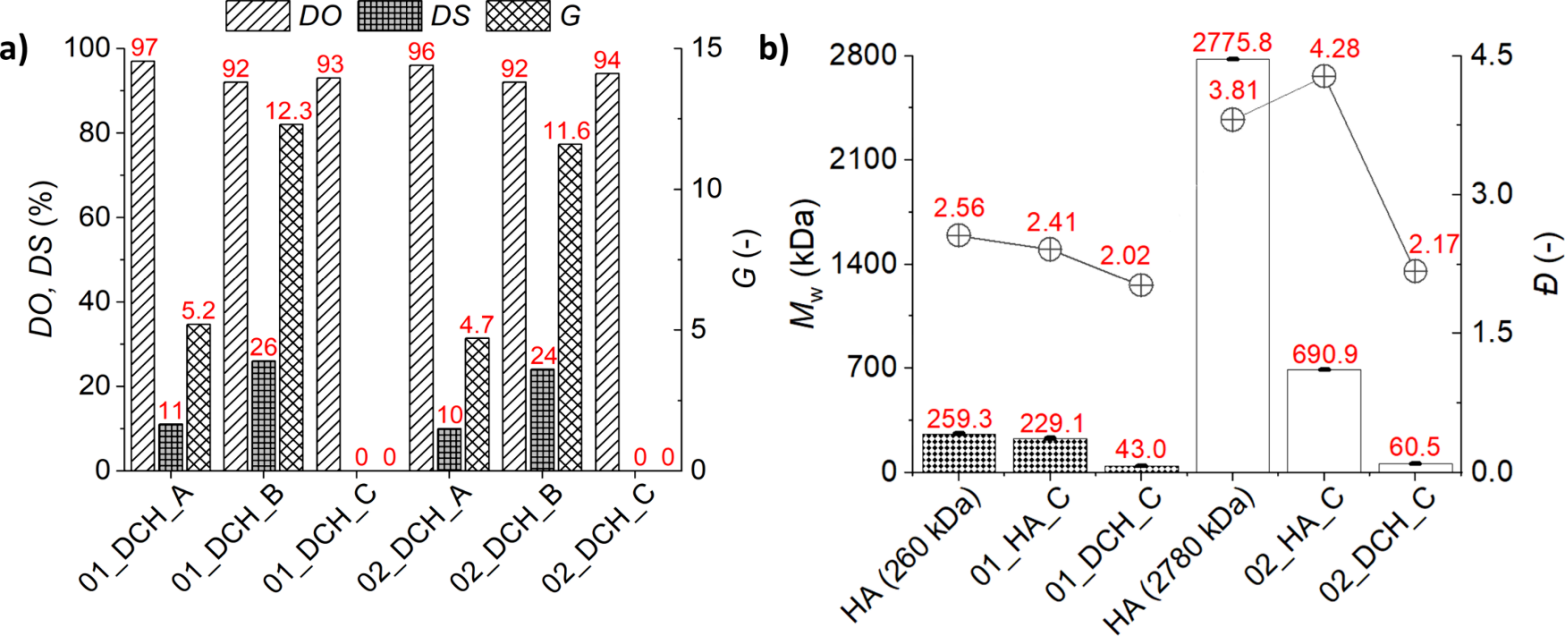
(a) Degree of oxidation (DO), degree of substitution (DS), and grafting (*G*) of DCH by pNIPAM determined from ^1^H NMR spectra. (b) Weight-average molecular weight (*M*_w_) and dispersity (*Đ*, hollow crossed points; line was added for the samples from the same source material to guide the eye) of the two source HA samples and their changes during individual reaction steps.

Signals of bound pNIPAM are very visible in the 01_DCH_A and B spectra. The assignment is based on previous reports,^39^ and signals are marked by Greek letters α–δ. There are no other signals that could be caused by degradation of pNIPAM during the oxidations. Grafted pNIPAM thus remained intact during the whole process.

Grafting (*G*) of the pNIPAM was established from a comparison of the integral intensity of signals Hα of pNIPAM (6 × H) and H5 of DCH (1 × H), and DS was estimated based on eq 1, assuming DP_pNIPAM_ = 48 (eq 2). The *G* in series A was around 5, which corresponds to the DS of ∼10% (Figure 3a). The *G* and DS values of both samples from series B were approximately 2.4× higher (*G* = 12, DS = 25%) compared with those from series A; see Figure 3a. This is caused by different reaction ratios of HA:pNIPAM 1:1 (series A) and 1:2 (series B); see Table 1.

#### 3.1.1 Decrease of Molecular Weight during DCH-pNIPAM Preparation

The decrease in the weight-average molecular weight *M*_w_ during modifications was determined on series C samples prepared without pNIPAM to avoid possible column clogging at higher temperatures. Results are compared to those of source HA (Figure 3b).

After the simulated pNIPAM conjugation reaction, the *M*_w_ of the 01_HA_C sample decreased only by 12% and the dispersity *Đ* by 6% compared to the source HA (260 kDa). However, the *M*_w_ of the 02_HA_C sample, based on high-*M*_w_ HA (*M*_w_ = 2780 kDa), literally plunged by 75% while *Đ* increased by 12%. The use of high-*M*_w_ HA thus brings about limited benefits. The decrease of *M*_w_ is likely caused by acidic hydrolysis of glycosidic bonds in HA during a rather long reaction (5 days) at pH 6.5,^40^ because of a higher probability of random hydrolysis of longer HA chains.

The oxidation step resulted in a further decrease of *M*_w_ by 80–90% in both series. The *Đ* decreased to 2.02 (series 01) and 2.17 (series 02). Degradation is similar to that reported previously.^23^ Nevertheless, despite the observed decrease in molecular weight, the obtained DCH conjugates had *M*_*w*_ values of 43.0 kDa (01) and 60.5 kDa (02), which are still suitable for nanogel preparation.

### 3.2 Thermoresponsive Behavior of DCH-pNIPAM Conjugates

The LCSTs of DCH-pNIPAM conjugates were estimated based on changes in their storage modulus (*G*′). The temperature dependence of *G*′ of all DCH-pNIPAM conjugates and blank samples is given in Figure 4a. While a notable change of *G*′ in DCH-pNIPAM conjugate samples occurred between 31 and 33 °C, blank nonthermoresponsive samples showed no significant changes of *G*′ in this temperature range. The changes of *G*′ are thus caused by the sol–gel transition of the conjugated pNIPAM. The LCSTs of all samples are similar to free pNIPAM (LCST = 32.3 °C).^39,41^ No clear trends in LCST values could be determined among the samples, as samples 01_DCH_A, 02_DCH_A, and 02_DCH_B have LCSTs between 31.1 and 31.6 °C, while the LCST of 01_DCH_B is somewhat higher (33 °C). Nevertheless, all conjugates underwent sol–gel transitions well below body temperature and are thus suitable for intended applications.

**Figure 4:**
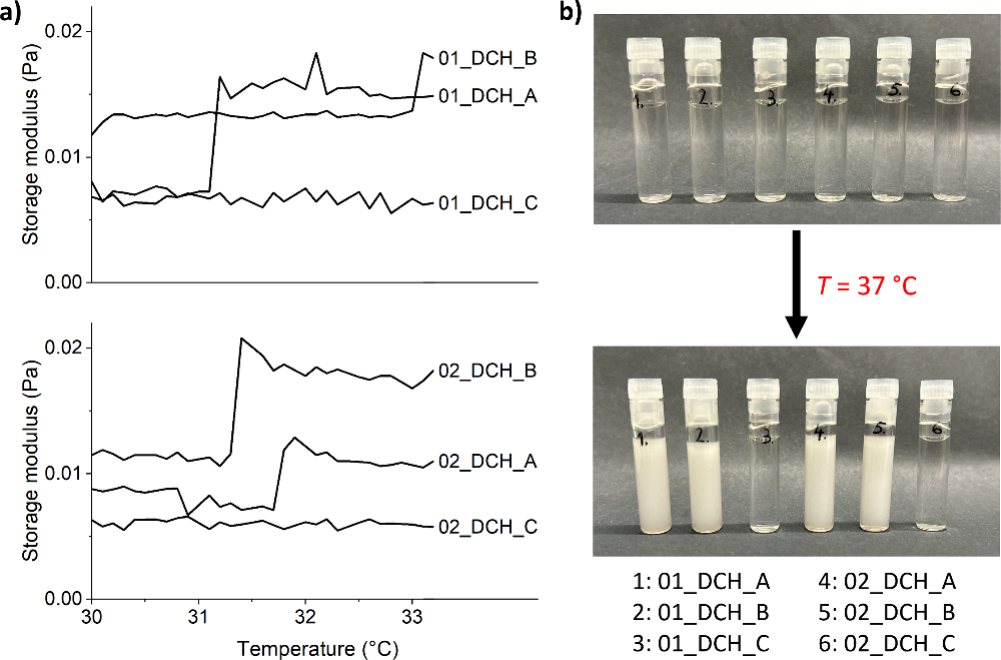
(a) Temperature dependence of storage modulus of all samples dissolved in UPW (20 mg/mL) and (b) visualization of turbidity change of these samples at 37 °C.

To further illustrate the changes occurring during the sol–gel transition, macroscopic observations of conjugate solutions were performed (Figure 4b). While all samples formed clear solutions at laboratory temperature (below LCST), samples from series A and B showed white turbidity after raising the temperature to 37 °C. In comparison, no turbidity was observed in samples from series C.

Next, the hydrodynamic diameters (*d*_H_, Figure 5a), ζ-potentials (Figure 5b), and polydispersity indexes (PDI, Table S1) of DCH-pNIPAM conjugates and series C samples were measured at 25 and 37 °C. All conjugates form colloidal particles of *d*_H_ between 200 and 600 nm at both temperatures. While the *d*_H_ of blank samples decreased when measured at 37 °C, the *d*_H_ values of DCH-pNIPAM conjugates did not change significantly after heating. Nevertheless, there are distinct differences among individual samples. Samples from series B with a higher DS of pNIPAM had generally lower *d*_H_ compared to corresponding samples from series A at both tested temperatures. Similarly, higher *M*_w_ of the source HA (series 02) resulted in lower *d*_H_ compared to the corresponding samples in series 01. The smallest nanoassemblies were thus obtained for conjugate 02_DCH_B, which combines higher *M*_w_ and higher *DS*. Moreover, all thermoresponsive samples show decreased PDI values above LCST, meaning that the uniformity of the DCH-pNIPAM conjugates is higher after the transition (Table S1).

**Figure 5:**
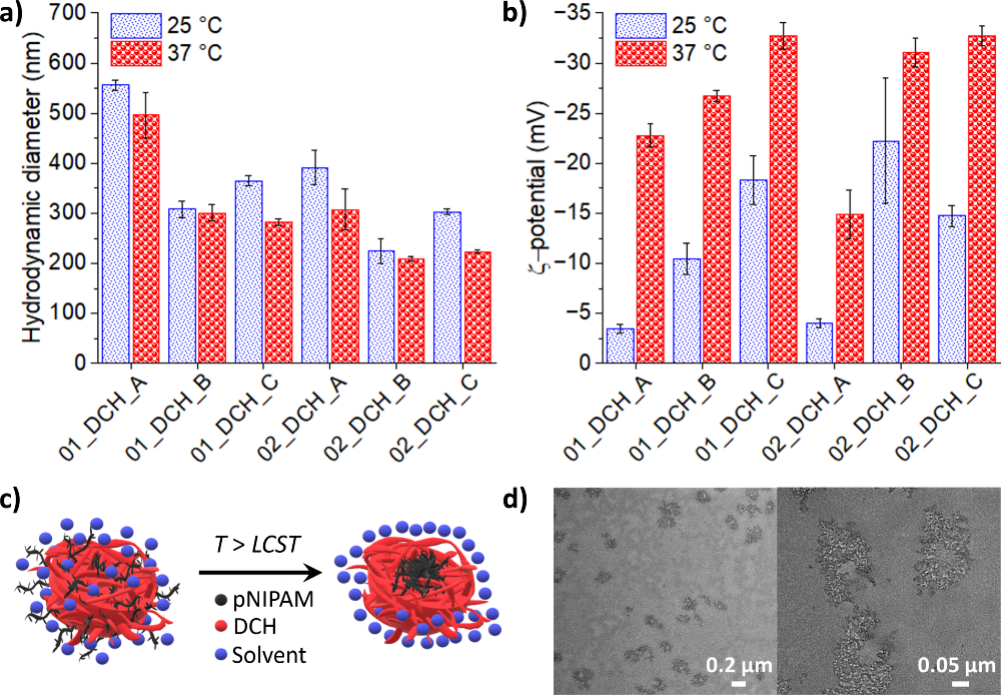
(a) Hydrodynamic diameter *d*_H_ and (b) ζ-potential of all samples measured in UPW at 25 and 37 °C. (c) Illustration of the reorganization process within the structure of DCH-pNIPAM colloidal particles triggered by increasing the temperature above LCST. (d) TEM image of 02_DCH_A dried at 25 °C at lower (left) and higher (right) resolution.

Regarding the trends in ζ-potentials, the absolute values of ζ-potentials are generally influenced by the particle size, the presence of charged functional groups in the particle, and the temperature of the measurement. The temperature dependence is likely responsible for the observed increase of ζ-potentials of all samples after heating to 37 °C.^42^ Regarding the presence of charged groups, the more −COO^–^ groups available in prepared nanoassemblies, the more negative the ζ-potential should be (assuming similar particle sizes). In other words, the absolute values of ζ-potentials of series C samples, which contain the highest number of unsubstituted −COO^–^ groups, should be higher than those in series A and particularly in series B samples with the highest DS. Yet, this is not the case, particularly for the 02_DCH_B sample, which has the same ζ-potential as the unsubstituted 02_DCH_C sample of similar size. A possible explanation lies in a reorganization of the inner structure of the colloidal particles formed by DCH-pNIPAM conjugates. This is allowed by the high flexibility of DCH chains, caused by C2–C3 bond scission during POX.^43^ When grafted pNIPAM chains undergo a conformational change above LCST, their hydrophobicity dramatically increases. The hydrophobic effect then initiates their reorientation toward the core of the particles, while unsubstituted negatively charged −COO^–^ groups will be pushed closer to the particle surface; see Figure 5c. This process may be further enhanced by the mutual repulsion of unsubstituted COO^–^ groups in the DCH chains. This theory is supported by further evidence, as shown in the following sections.

TEM images of DCH-pNIPAM conjugates show well-separated amorphous particles about 100–300 nm in diameter; see Figure 5d. Observed structures correspond to polymeric colloidal particles that collapsed during drying. Upon closer inspection (Figure 5d, right), it seems that assemblies are formed from smaller structures of several nanometers in size. All colloidal particles also seem to possess a central “cavity”, which indicates micelle-like structures.

### 3.3 Preparation of PhPt-Loaded DCH-pNIPAM Nanogels

As a next step, DCH-pNIPAM conjugates were loaded with PhPt, a next-generation platinum anticancer drug with a nonclassical mechanism of action.^35,44^ PhPt was selected because (a) its biological efficacy is known to be enhanced by various carriers,^45,46^ (b) it is a positively charged monodentate platinum­(II) complex, which is ideal for DCH, as one of the −COO^–^ groups can substitute the chloride ligand, while the second one counters the positive charge of the PhPt complex, (c) its large hydrophobic aromatic moiety could, potentially, stabilize the structure of the colloidal particles by noncovalent interactions, e.g., by π–π stacking, which may cross-link the DCH-pNIPAM conjugates into nanogels and suppress the possible internal reorganization of particles above LCST. Although speculative, the hypothesis about the formation of nanogels is in good agreement with the obtained results; see below. DCH-pNIPAM particles loaded with PhPt are thus termed nanogels in the following text to simplify the discussion.

Two PhPt-loading protocols were tested, differing mainly in the pH of the activated PhPt, which was not adjusted (series P1) or set to 7 before loading (P2); see section 2.3. The 01_DCH_A sample was excluded from this study due to its very large *d*_H_.

Initially, the *d*_H_ of PhPt-loaded DCH-pNIPAM nanogels was measured in PBS at pH 7.4. These conditions were selected to better represent the environment of the human body (Figure 6a). Below LCST, all nanogels had smaller *d*_H_ values than the corresponding DCH-pNIPAM conjugates without PhPt (see Figure 5a), indicating significantly more “compressed” structures. Note, however, that this may be a result of a change of dispersion medium (UPW vs PBS), and a direct comparison thus may be misleading.

**Figure 6:**
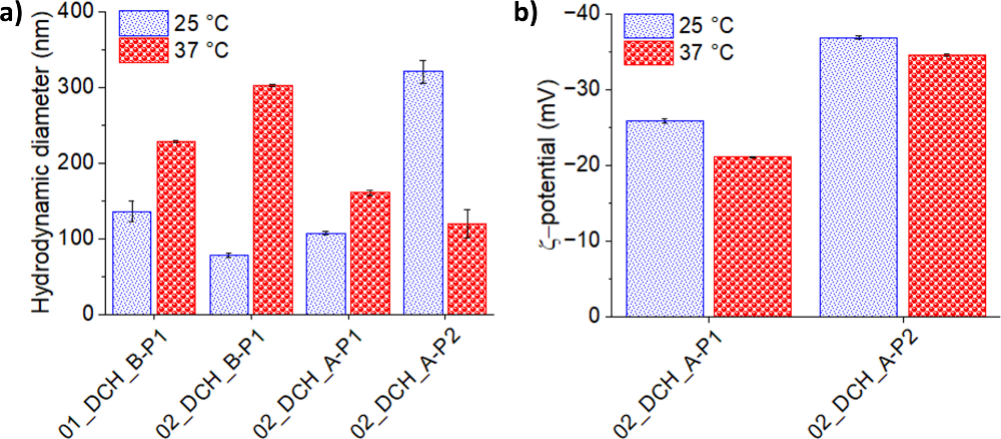
(a) Hydrodynamic diameter *d*_H_ and (b) ζ-potential of PhPt-loaded DCH-pNIPAM conjugates measured in PBS 7.4.

Interestingly, temperature-induced changes in *d*_H_ of PhPt-loaded nanogels were much larger than those in neat DCH-pNIPAM conjugates. They also differed significantly among samples; see Figure 6a. Series P1 nanogels experienced a significant increase in *d*_H_ above LCST, while a decrease in *d*_H_ was observed in the P2 nanogel. The largest overall changes in *d*_H_ were observed for the 02_DCH_B–P1 (3.75 times increase in *d*_H_, from ∼80 to ∼300 nm) and 02_DCH_A_P2 nanogels (2.75 times decrease in *d*_H_, from ∼320 to ∼120 nm). In contrast, 02_DCH_A-P1 showed the lowest change of only about a 50% increase in *d*_H_. The ζ-potential measurement performed for 02_DCH_A_P1 and 02_DCH_A_P2 nanogels did not offer any clues to explain the differences, showing only a minor decrease in absolute values of ζ-potential above LCST; see Figure 6b. Most importantly, however, the PDI values of all samples decreased dramatically above the LCST, i.e., from 0.5–0.7 to 0.12–0.14 in the series P1 nanogels and from 0.9 to 0.4 for the series P2 nanogel (Table 2). Polydisperse colloidal systems present at laboratory temperature thus spontaneously reassemble into nanogels with significantly narrower size distribution at body temperature, which is highly beneficial for drug delivery applications.^47^

**Table 2:** The Polydispersity Index (PDI) of Series P1 and P2 Samples Is below and above the LCST

code	PDI at 25 °C (−)	PDI at 37 °C (−)
01_DCH_B-P1	0.76 ± 0.07	0.14 ± 0.01
02_DCH_B-P1	0.52 ± 0.03	0.13 ± 0.01
02_DCH_A-P1	0.55 ± 0.08	0.12 ± 0.01
02_DCH_A-P2	0.90 ± 0.10	0.40 ± 0.06

To explain the different behavior of the nanogels, TEM micrographs of 02_DCH_A-P2 nanogels dried below and above the LCST were obtained in an attempt to capture any temperature-related changes in their structure (Figure 7). The 02_DCH_A-P2 sample prepared by drying below the LCST contained particles larger than those of the heated sample, as seen in Figure 7a,c. This is in agreement with the DLS results discussed above. Moreover, the darker shade of the nanogel particle in Figure 7b indicates that electron-dense platinum atoms are rather uniformly spread within the particles below the LCST. However, in the nanogel dried at 40 °C (Figure 7d), the electron-dense PhPt is found mostly on its surface. The 02_DCH_A-P2 nanogels thus likely underwent internal reorganization, similar to that discussed for neat DCH-pNIPAM conjugates in section 3.2. The underlying reason is unclear, but we can speculate that increasing the pH of the PhPt mixture before loading may lead to the formation of a stable PhPt hydroxo complex. Because platinum­(II) hydroxo complexes are rather inert,^48^ the substitution of the −OH ligand by a −COO^–^ group of DCH_pNIPAM thus may be suppressed. Consequently, PhPt in the 02_DCH_A-P2 nanogels could be held within particles mostly by ionic interactions, which may not prevent the rearrangement of nanogels above the LCST that is initiated by the sol–gel transition of pNIPAM discussed in section 3.2 (Figure 5c). This explains different trends in the *d*_H_ of P1 and P2 series nanogels and is supported by the results of drug release studies; see below.

**Figure 7:**
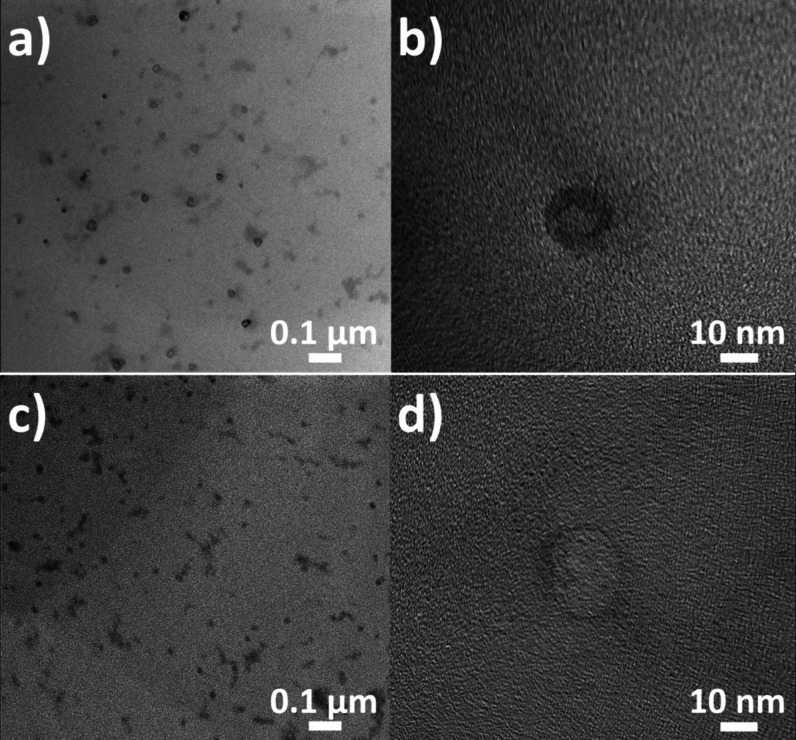
TEM images of 02_DCH_A-P2 sample dried (a, b) at 25 °C and (c, d) at 40 °C.

The TEM micrographs also further support the formation of nanogels after PhPt loading, as expected based on other polysaccharide-based platinum drug carriers.^17,38,49^ While in solution, PhPt-loaded nanogels have a hydrodynamic diameter between 80 and 300 nm, but the TEM images in Figure 7 show spherical particles with approximately 20 nm in size. This indicates significant swelling of the species in solution, which is one of the main characteristics of nanogel systems.^38^ In comparison, dried free carriers (Figure 5) have amorphous, nonspherical shapes of 200+ nm in size. This is comparable to their *d*_H_ in the solution, meaning that their swelling is limited, and their structure is not preserved during drying. The observed behavior thus provides a strong indication of nanogel formation during PhPt loading.

### 3.4 Phenanthriplatin Release from DCH-pNIPAM Nanogels

The size of the drug carriers is paramount to their biological efficacy. The carriers with smaller dimensions, usually below 100–150 nm, are generally considered to be the best,^50,51^ as they can relatively easily penetrate tumors, where they accumulate due to enhanced permeability and retention effect.^47^ Hence, two samples with the lowest *d*_H_, namely, 02_DCH_A-P1 and 02_DCH_A-P2, were chosen for the drug release study and biological evaluation. These were compared with a nonthermoresponsive oxidized hyaluronan sample (DCH), a nonoxidized thermoresponsive 02_HA_A sample, and an unmodified HA sample (HA), which were loaded by PhPt under the same conditions as the P2 sample. The direct comparison between samples allows us to demonstrate the advantages of the current approach.

The first major difference between the samples can be observed in the loading effectiveness (Table 3), which directly relates to their carrier capacity. The highest effectiveness was observed for the 02_DCH_A-P1 sample, with nearly 95% of PhPt still conjugated to the carrier even after purification. Both oxidized P2 samples reached a loading efficiency of around 80%, probably due to higher losses during the purification caused by faster drug release, as seen below. High loading efficacies of all oxidized species are in agreement with the expected availability of drug-binding sites due to the absence of steric hindrance by pNIPAM, as discussed in the introduction.

**Table 3:** Loading Efficiency of the Samples from Figure 8

code	loading efficiency (%)
HA-P2	63.8 ± 0.6
02_HA_A-P2	27.8 ± 0.6
DCH_P2	80.0 ± 0.7
02_DCH_A-P1	93.8 ± 0.7
02_DCH_A-P2	81.3 ± 0.9

On the contrary, the unoxidized samples showed significantly lower drug loading efficacies. The neat HA (HA-P2 sample) could hold only 64% of the loaded PhPt after purification, likely due to the limited number of drug-binding sites at C6 and fast drug release rates. When HA was modified by pNIPAM (02_HA_A-P2), the amount of drug-binding sites decreased further and steric hindrance increased, resulting in only 28% of drug loading efficacy. Selective oxidation thus provides a significant improvement in drug loading efficiency, particularly compared to that of thermoresponsive HA.

Drug release profiles of all samples are given in Figure 8 (PBS, pH 7.4, 37 °C). Note that the initial burst release of the PhPt present in all samples, corresponding to 15–20% of the carried drug, is probably caused by the sonication employed for better homogenization before the release experiment.

**Figure 8:**
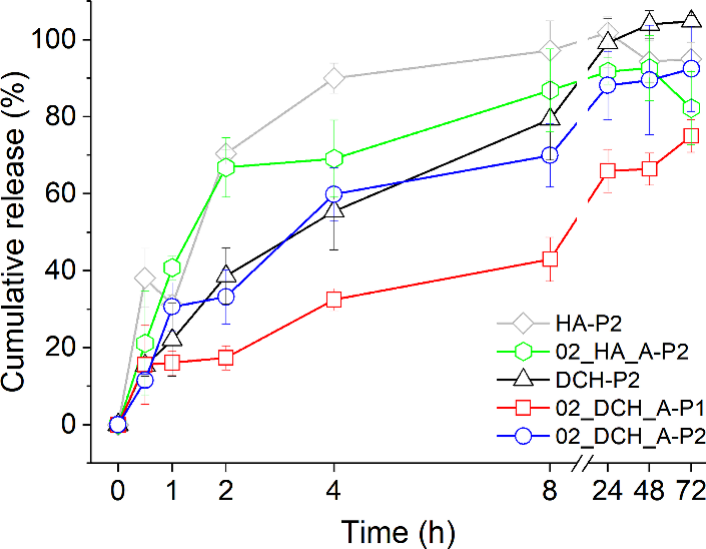
Cumulative release of PhPt from 02_HA_A-P2, 02_DCH_A-P1, and 02_DCH_A-P2 nanogels and nonthermoresponsive HA-P2 and DCH-P2 samples in PBS 7.4 at 37 °C.

The fastest overall drug release is observed for both unoxidized samples, which released about 70% of the loaded PhPt in 2 h. In comparison, both oxidized P2 samples (02_DCH_A-P2, DCH-P2) released only 35% of the loaded drug within 2 h, thus demonstrating the benefits of selective oxidation. Insignificant differences in drug release rates between 02_DCH_A-P2 and DCH-P2 support our assumption about the predominantly ionic binding of PhPt within P2 samples and indicate the negligible role of pNIPAM substitution in these samples due to suspected rearrangement of the particles above LCST. On the other hand, a much slower release from 02_DCH_A-P1 indicates a significant impact of pNIPAM substitution when particle rearrangement is suppressed. For illustration, while only about 40% of PhPt is released from 02_DCH_A-P1 after 8 h, about 70% is released from each of the 02_DCH_A-P2 or DCH-P2 samples, and release from HA-P2 and 02_HA_A-P2 is essentially finished. Nearly half of the drug is still bound in the 02_DCH_A-P1 nanogel, even after 24 h.

The selective oxidation, drug loading protocol, and presence of pNIPAM thus have a significant impact on drug release kinetics. Additional optimization of the loading procedure may further improve the drug release profiles.

### 3.5 Biological Evaluation

To date, there have been only a few reports on the cytotoxic activity of PhPt-carrier conjugates in cancer cell lines. De Luca *et al*. reported on the effect of the cytotoxic activity of PhPt-loaded wireframe DNA origami nanostructures in cancer cell lines.^52^ Nanomolar quantities of the ball-like origami nanostructure loaded with this drug decreased viability in the cisplatin-resistant breast adenocarcinoma cell line MCF-7 to 33% while being ineffective on the other tested cancer cell lines. Chen *et al.* prepared a reduction-sensitive polymer as a carrier for PhPt prodrug, showing improved anticancer efficacy and accumulation in tumors compared to CP and free PhPt.^53^ Czapar *et al.* encapsulated PhPt in the cavity of tobacco mosaic virus.^45^ The resulting conjugate showed 30–50% higher cytotoxicity toward breast and ovarian carcinoma *in vitro* than free PhPt and improved tumor accumulation. In one of our previous studies,^54^ we have reported data that suggests a possible synergistic effect of dialdehyde cellulose-cross-linked hydrogels combined with PhPt on the killing of human alveolar adenocarcinoma cell line A549.

Here, we further elaborate on the intriguing characteristics of PhPt, highlighting the potential of PhPt-loaded nanogels for anticancer drug delivery applications. The *in vitro* biological evaluation of 02_DCH_A-P1 and P2 samples was performed on human ovarian cancer cell line A2780 and its cisplatin-resistant subline A2780/CP. Cells were treated with compounds at concentrations ranging from 0 to 25 μM for 48 h. The cytotoxicity of PhPt-bearing nanogels was compared with that of free PhPt in equimolar concentration of the drug. The obtained differences thus represent the impact of nanogel formulations. The IC_50_ values (in μM) are given in Figure 9 and in Table 4. Moreover, free 02_DCH_A conjugate (the free carrier) was tested as well, exhibiting no significant toxicity across the concentration range of 0–25 μM in both cell lines and at three time points (24, 48, and 72 h), indicating its excellent biocompatibility. For details, see Figure S1.

**Figure 9:**
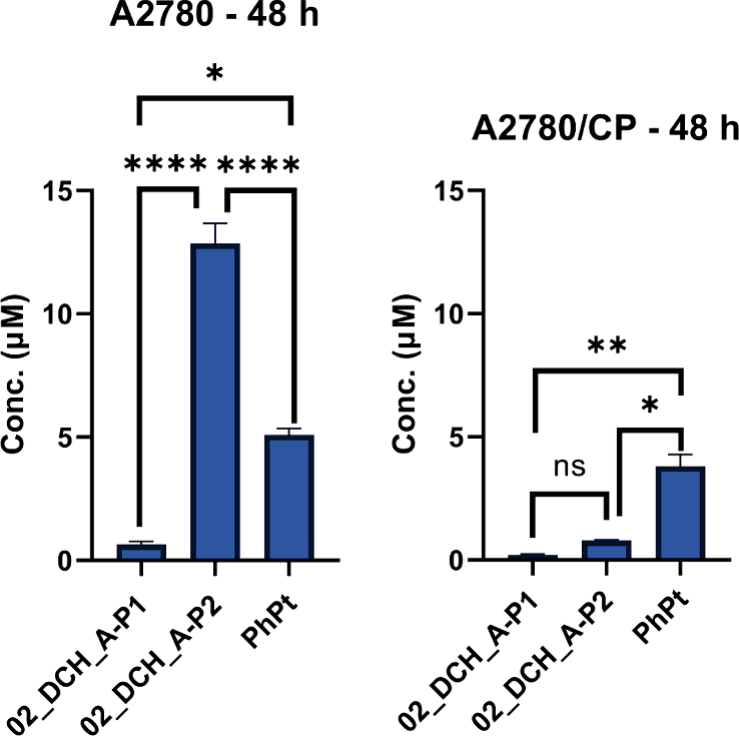
The IC_50_ values (in μM) for each cell line were determined after a 48 h incubation period. Error bars indicate ± SEM. Statistical analysis was conducted using ANOVA, followed by Tukey’s multiple comparison test.

**Table 4:** Effects of 02_DCH_A-P1, 02_DCH_A-P2, and PhPt on Cell Viability Expressed as Absolute IC_50_ Concentrations^a^

	IC_50_ (μM)		
cell line	02_DCH_A-P1	02_DCH_A-P2	PhPt
A2780	0.64 ± 0.13	12.86 ± 0.81	5.09 ± 0.27
A2780/CP	0.21 ± 0.04	0.79 ± 0.03	3.81 ± 0.48

a Values represent the average of four independent measurements ± SEM.

Drug-loaded nanogel samples exhibited distinctly different effects on the cell viability. The 02_DCH_A-P1 demonstrated significantly higher anticancer efficacy compared to free PhPt, being 8 times more effective in A2780 cells and more than 18 times more effective in A2780/CP cells. Conversely, the cytotoxic effect of the 02_DCH_A-P2 sample was markedly lower than that of P1, especially in A2780 cells. In these cells, its *in vitro* cytotoxic activity was more than twice as low as free PhPt and 20 times lower than the 02_DCH_A-P1 nanogel. The decrease in efficacy is consistent with the suspected passivation of PhPt in the 02_DCH_A-P2 nanogel, as discussed above.

Compared to the parental A2780 cell line, the 02_DCH_A-P1 and 02_DCH_A-P2 nanogels exhibited over 3-fold and almost 17-fold enhancements in efficacy, respectively, in CP-resistant A2780/CP cells. Interestingly, free PhPt was “only” 1.3 times more effective in A2780/CP cells than in the parental A2780 cells. This effect may be attributed to a distinct mode of action compared to cisplatin, as PhPt causes cell cycle arrest through the inhibition of topoisomerase II.^36^ Notably, its efficacy seems to be greatly enhanced by the conjugation of PhPt to a thermoresponsive carrier.

Next, scratch assays were performed on selected cell lines; see Figure 10. All samples were tested at their previously established IC_25_ concentrations, rather than equimolar doses, to specifically evaluate the influence of compounds on cell migration and not on cell proliferation. PhPt was found to dramatically decrease the cellular motility of both ovarian cancer cell lines by approximately 17-fold. When incorporated into nanogels, the migrastatic properties of PhPt were significantly reduced, probably due to the slower onset of the cytotoxic effect caused by gradual drug release. Still, both nanogels loaded with PhPt exhibited statistically significant inhibition of cellular migration compared with untreated cells, as illustrated in Figure 10 and Table 5. Notably, the 02_DCH_A-P2 exhibited comparable efficacy to free PhPt in A2780 cells. In contrast, Baribeau *et al.* reported that after 48 h of CP treatment, A2780 cells still retained 50% of their original migration capacity when compared to the vehicle group, while in A2780/CP cells, no effect of CP treatment on cell migration was observed when compared with untreated cells.^55^

**Figure 10:**
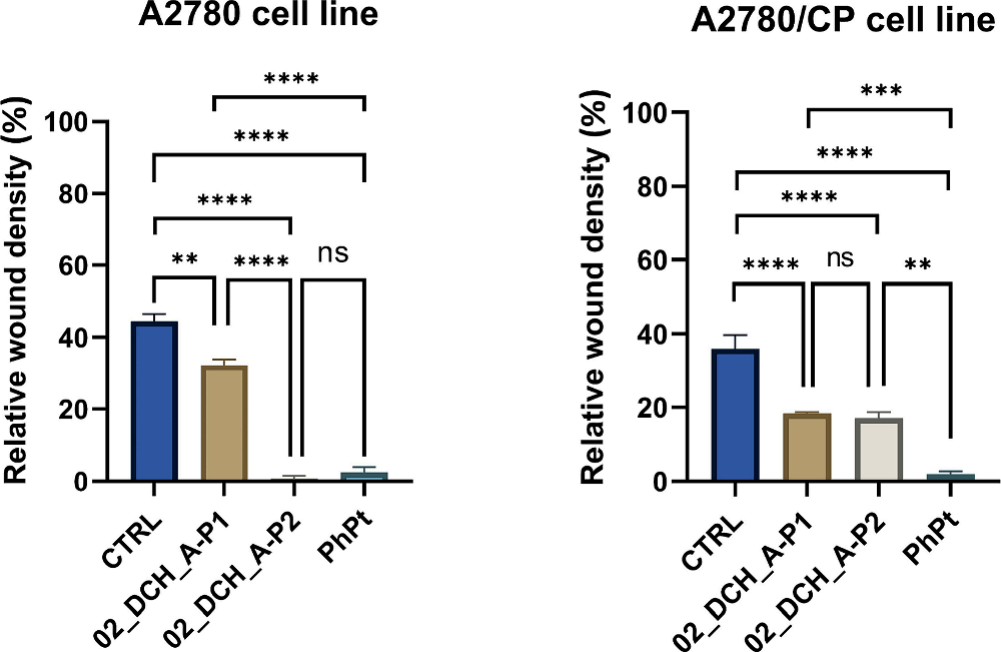
Inhibition analysis of cell migration assessed by a scratch assay in A2780 and A2780/CP cells following treatment with 02_DCH_A-P1 and 02_DCH_A-P2 compared to PhPt and untreated control cells. Relative wound density was measured after 48 h of treatment with the respective compounds. Bars represent the mean ± SEM, *n* = 6, **p < 0.001, ***p < 0.0001. Statistical analysis was performed by one-way ANOVA followed by Tukey’s test.

**Table 5:** Effects of 02_DCH_A-P1, 02_DCH_A-P2, and PhPt on Cell Migration Expressed as Changes in Relative Wound Density after 48 h of Exposure^a^

	relative wound density (%)			
cell line	CTRL	02_DCH_A-P1	02_DCH_A-P2	PhPt
A2780	44.5 ± 2.0	32.3 ± 1.6	0.79 ± 0.74	2.5 ± 1.4
A2780/CP	36.0 ± 3.6	18.4 ± 0.3	17.1 ± 1.6	2.1 ± 0.7

a Values represent the average of six independent measurements ± SEM.

## 4 Conclusions

In this study, thermoresponsive nanogels were successfully prepared by conjugation of poly­(*N*-isopropyl acrylamide) (pNIPAM) to hyaluronic acid (HA), followed by selective oxidation of HA to 2,3-dicarboxy hyaluronate (DCH). Although a decrease in *M*_w_ was observed during the process, no degradation of conjugated pNIPAM occurred, confirming the usability of the employed method. The lower critical solution temperature (LCST) of the DCH-pNIPAM conjugates was between 31 and 33 °C, which is comparable to the LCST of free pNIPAM (32.3 °C). The hydrodynamic diameters (*d*_H_) and ζ-potentials of the DCH-pNIPAM conjugates were affected by two parameters: the degree of substitution (DS) of pNIPAM and *M*_w_ of the source HA. Both higher DS and higher *M*_w_ of the source HA led to a decrease in *d*_H_ and an increase in absolute values of ζ-potential. The DCH-pNIPAM conjugates formed colloidal particles that underwent reorganization upon heating above the LCST. In such a case, hydrophilic unsubstituted DCH units formed an outer shell, while hydrophobic pNIPAM chains are most likely located in the core. This is, to the best of our knowledge, the first time such behavior was described for a pNIPAM-decorated hydrophilic polymer.

Subsequently, PhPt was loaded into the conjugates, which self-assembled into nanogels characterized by a narrow size distribution and low polydispersity above the LCST. Two drug loading protocols were used. Adjustment of the pH value of PhPt solution to pH = 7.0 before its loading most likely led to the passivation of PhPt, preventing its covalent conjugation and causing rapid release from 02_DCH_A-P2 nanogels. In comparison, the PhPt-loaded 02_DCH_A-P1 nanogel exhibited reduced drug-release rates and optimal size for the drug-delivery carrier. Compared to unoxidized pNIPAM-substituted HA, the 02_DCH_A-P1 showed more than three times higher PhPt loading efficiency and significantly slower release. This demonstrates the advantage of selective oxidation after the pNIPAM binding.

We further evaluated the *in vitro* cytotoxicity and migrastatic potential of the 02_DCH_A-P1 and 02_DCH_A-P2 nanogels, comparing their effects to those of free PhPt. While the free carrier was nontoxic in the given concentration range, the nanogel samples demonstrated distinctly different effects on cell viability based on their characteristics. The 02_DCH_A-P1 sample exhibited significantly higher anticancer efficacy than free PhPt, being 8 times more effective in A2780 cells and 18 times more effective in A2780/CP cells. Contrarily, the 02_DCH_A-P2 sample showed markedly lower cytotoxicity, emphasizing the importance of carrier design on its biological properties. Notably, both the 02_DCH_A-P1 and 02_DCH_A-P2 nanogels exhibited over three and 16 times higher efficacy, respectively, in CP-resistant A2780 cells compared to the parental A2780 cell line, further strengthening the effect observed in free PhPt, which was 1.3 times more effective in cisplatin-resistant A2780/CP cells than in the parental A2780 cells. Our findings thus demonstrate that binding to nanogels enhances the therapeutic potency of the PhPt and significantly increases its cell-killing activity in cisplatin-resistant ovarian cancer cells.

Scratch assays were also conducted on selected cell lines to assess the impact of neat PhPt and its nanogel counterparts on cell migration. Our data demonstrated that PhPt significantly decreased the cellular motility of both ovarian cancer cell lines. Additionally, all PhPt-loaded nanogels maintained a statistically significant inhibition of cellular migration compared with untreated cells, indicating that the migrastatic properties of PhPt were partially preserved, albeit with a significant reduction upon incorporation into nanogels.

In conclusion, we report the synthesis of thermoresponsive PhPt-loaded nanogels exhibiting significant potential in cancer therapy, as illustrated by their efficacy against ovarian cancer cells and their cisplatin-resistant variant. Moreover, thermoresponsive behavior is particularly beneficial as it allows for injection into affected tissues, such as post-tumor resection sites, potentially aiding in the elimination of residual cancerous cells. Our findings suggest that incorporating PhPt into nanogels not only preserves but also enhances its properties, offering a promising strategy to address the challenges of drug resistance in cancer treatment.

## Supplementary Material



## Data Availability

Data will be made available on request.

## Notes

Polydispersity of the samples and cytotoxicity of free carrier toward A2780 and A2780/CP cell lines (PDF)

## References

[1] Fraser J. R. E., Laurent T. C., Laurent U. B. G. (1997). Hyaluronan: Its Nature, Distribution,
Functions and Turnover. J. Int. Med..

[2] Cyphert J. M., Trempus C. S., Garantziotis S. (2015). Size Matters: Molecular Weight Specificity
of Hyaluronan Effects in Cell Biology. Int.
J. Cell Biol..

[3] Girish K. S., Kemparaju K. (2007). The Magic Glue Hyaluronan and Its Eraser Hyaluronidase:
A Biological Overview. Life Sci..

[4] Knopf-Marques H., Pravda M., Wolfova L., Velebny V., Schaaf P., Vrana N. E., Lavalle P. (2016). Hyaluronic Acid and Its Derivatives
in Coating and Delivery Systems: Applications in Tissue Engineering,
Regenerative Medicine and Immunomodulation. Adv. Healthc. Mater..

[5] Toole B. P. (2004). Hyaluronan:
From Extracellular Glue to Pericellular Cue. Nat. Rev. Cancer.

[6] Turley E. A., Noble P. W., Bourguignon L. Y. W. (2002). Signaling Properties of Hyaluronan
Receptors. J. Biol. Chem..

[7] Dosio F., Arpicco S., Stella B., Fattal E. (2016). Hyaluronic Acid for
Anticancer Drug and Nucleic Acid Delivery. Adv.
Drug Delivery Rev..

[8] Harrer D., Sanchez Armengol E., Friedl J. D., Jalil A., Jelkmann M., Leichner C., Laffleur F. (2021). Is Hyaluronic Acid
the Perfect Excipient
for the Pharmaceutical Need?. Int. J. Pharm..

[9] Khazaei Z., Namayandeh S. M., Beiranvand R., Naemi H., Bechashk S. M., Goodarzi E. (2021). Worldwide
Incidence and Mortality of Ovarian Cancer
and Human Development Index (HDI): GLOBOCAN Sources and Methods 2018. J. Prev. Med. Hyg..

[10] du
Bois A., Baert T., Vergote I. (2019). Role of Neoadjuvant Chemotherapy
in Advanced Epithelial Ovarian Cancer. J. Clin.
Oncol..

[11] Raudenska M., Balvan J., Fojtu M., Gumulec J., Masarik M. (2019). Unexpected
Therapeutic Effects of Cisplatin. Metallomics.

[12] Zhang C., Xu C., Gao X., Yao Q. (2022). Platinum-Based Drugs for Cancer Therapy
and Anti-Tumor Strategies. Theranostics.

[13] Huang D., Savage S. R., Calinawan A. P., Lin C., Zhang B., Wang P., Starr T. K., Birrer M. J., Paulovich A. G. (2021). A Highly
Annotated Database of Genes Associated with Platinum Resistance in
Cancer. Oncogene.

[14] Nunes M., Bartosch C., Abreu M. H., Richardson A., Almeida R., Ricardo S. (2024). Deciphering the Molecular
Mechanisms
behind Drug Resistance in Ovarian Cancer to Unlock Efficient Treatment
Options. Cells.

[15] Bokatyi A. N., Dubashynskaya N. V., Skorik Y. A. (2024). Chemical Modification of Hyaluronic
Acid as a Strategy for the Development of Advanced Drug Delivery Systems. Carbohydr. Polym..

[16] Cai S., Xie Y., Bagby T. R., Cohen M. S., Forrest M. L. (2008). Intralymphatic Chemotherapy
Using a Hyaluronan-Cisplatin Conjugate. J. Surg.
Res..

[17] Ohta S., Hiramoto S., Amano Y., Sato M., Suzuki Y., Shinohara M., Emoto S., Yamaguchi H., Ishigami H., Sakai Y., Kitayama J., Ito T. (2016). Production
of Cisplatin-Incorporating Hyaluronan Nanogels via Chelating Ligand-Metal
Coordination. Bioconjugate Chem..

[18] Quan Y. H., Kim B., Park J.-H., Choi Y., Choi Y. H., Kim H. K. (2014). Highly
Sensitive and Selective Anticancer Effect by Conjugated HA-Cisplatin
in Non-Small Cell Lung Cancer Overexpressed with CD44. Exp. Lung Res..

[19] Hintze V., Schnabelrauch M., Rother S. (2022). Chemical Modification of Hyaluronan
and Their Biomedical Applications. Front. Chem..

[20] Muir V. G., Burdick J. A. (2021). Chemically Modified
Biopolymers for the Formation of
Biomedical Hydrogels. Chem. Rev..

[21] Schanté C. E., Zuber G., Herlin C., Vandamme T. F. (2011). Chemical Modifications
of Hyaluronic Acid for the Synthesis of Derivatives for a Broad Range
of Biomedical Applications. Carbohydr. Polym..

[22] Fan X., Zhao X., Qu X., Fang J. (2015). pH Sensitive Polymeric
Complex of Cisplatin with Hyaluronic Acid Exhibits Tumor-Targeted
Delivery and Improved in Vivo Antitumor Effect. Int. J. Pharm..

[23] Münster L., Capáková Z., Humpolíček P., Kuřitka I., Christensen B. E., Vícha J. (2022). Dicarboxylated
Hyaluronate: Synthesis of a New, Highly Functionalized and Biocompatible
Derivative. Carbohydr. Polym..

[24] Münster L., Fojtů M., Capáková Z., Muchová M., Musilová L., Vaculovič T., Balvan J., Kuřitka I., Masařík M., Vícha J. (2021). Oxidized Polysaccharides
for Anticancer-Drug Delivery: What Is the Role of Structure?. Carbohydr. Polym..

[25] D’Este M., Eglin D., Alini M. (2014). A Systematic Analysis of DMTMM vs
EDC/NHS for Ligation of Amines to Hyaluronan in Water. Carbohydr. Polym..

[26] Capella V., Rivero R. E., Liaudat A. C., Ibarra L. E., Roma D. A., Alustiza F., Mañas F., Barbero C. A., Bosch P., Rivarola C. R., Rodriguez N. (2019). Cytotoxicity
and Bioadhesive Properties
of Poly-*N*-Isopropylacrylamide Hydrogel. Heliyon.

[27] Cooperstein M. A., Canavan H. E. (2013). Assessment of Cytotoxicity of (N-Isopropyl
Acrylamide)
and Poly­(N-Isopropyl Acrylamide)-Coated Surfaces. Biointerphases.

[28] Soriano
Pérez M. L., Funes J. A., Flores Bracamonte C., Ibarra L. E., Forrellad M. A., Taboga O., Cariddi L. N., Salinas F. J., Ortega H. H., Alustiza F., Molina M. (2023). Development
and Biological Evaluation of *p*NIPAM-Based Nanogels
as Vaccine Carriers. Int. J. Pharm..

[29] Barbier L., Protat M., Pipart P., Marcellan A., Tran Y., Hourdet D. (2023). Sol/Gel Transition
of Thermoresponsive
Hyaluronan: From Liquids to Elastic and Sticky Materials. Carbohydr. Polym..

[30] Atoufi Z., Kamrava S. K., Davachi S. M., Hassanabadi M., Saeedi Garakani S., Alizadeh R., Farhadi M., Tavakol S., Bagher Z., Hashemi Motlagh G. (2019). Injectable PNIPAM/Hyaluronic Acid
Hydrogels Containing Multipurpose Modified Particles for Cartilage
Tissue Engineering: Synthesis, Characterization, Drug Release and
Cell Culture Study. Int. J. Biol. Macromol..

[31] Atoufi Z., Kamrava S. K., Davachi S. M., Hassanabadi M., Saeedi Garakani S., Alizadeh R., Farhadi M., Tavakol S., Bagher Z., Hashemi Motlagh G. (2019). Injectable
PNIPAM/Hyaluronic Acid
Hydrogels Containing Multipurpose Modified Particles for Cartilage
Tissue Engineering: Synthesis, Characterization, Drug Release and
Cell Culture Study. Int. J. Biol. Macromol..

[32] Luckanagul J. A., Ratnatilaka Na Bhuket P., Muangnoi C., Rojsitthisak P., Wang Q., Rojsitthisak P. (2021). Self-Assembled
Thermoresponsive Nanogel
from Grafted Hyaluronic Acid as a Biocompatible Delivery Platform
for Curcumin with Enhanced Drug Loading and Biological Activities. Polymers.

[33] Solanki R., Bhatia D. (2024). Stimulus-Responsive Hydrogels for Targeted Cancer Therapy. Gels.

[34] Thodikayil A. T., Yadav A., Hariprasad P., Saha S. (2024). TEMPO-Oxidized Nanofibrillated
Cellulose as Potential Carrier for Sustained Antibacterial Delivery. Int. J. Biol. Macromol..

[35] Park G. Y., Wilson J. J., Song Y., Lippard S. J. (2012). Phenanthriplatin,
a Monofunctional DNA-Binding Platinum Anticancer Drug Candidate with
Unusual Potency and Cellular Activity Profile. Proc. Natl. Acad. Sci. U. S. A..

[36] Riddell I. A., Park G. Y., Agama K., Pommier Y., Lippard S. J. (2016). Phenanthriplatin
Acts as a Covalent Topoisomerase II Poison. ACS Chem. Biol..

[37] Vann K. R., Oviatt A. A., Osheroff N. (2021). Topoisomerase
II Poisons: Converting
Essential Enzymes into Molecular Scissors. Biochemistry.

[38] Münster L., Fojtů M., Muchová M., Latečka F., Káčerová S., Capáková Z., Juriňáková T., Kuřitka I., Masařík M., Vícha J. (2021). Enhancing
Cisplatin Anticancer Effectivity and Migrastatic Potential by Modulation
of Molecular Weight of Oxidized Dextran Carrier. Carbohydr. Polym..

[39] D’Este M., Alini M., Eglin D. (2012). Single Step Synthesis and Characterization
of Thermoresponsive Hyaluronan Hydrogels. Carbohydr.
Polym..

[40] Tømmeraas K., Melander C. (2008). Kinetics of Hyaluronan Hydrolysis
in Acidic Solution
at Various pH Values. Biomacromolecules.

[41] Chen J.-P., Leu Y.-L., Fang C.-L., Chen C.-H., Fang J.-Y. (2011). Thermosensitive
Hydrogels Composed of Hyaluronic Acid and Gelatin as Carriers for
the Intravesical Administration of Cisplatin. J. Pharm. Sci..

[42] Venditti, R. Temperature Effects on the Zeta Potential. In Encyclopedia of Microfluidics and Nanofluidics; Li, D. , Ed.; Springer US: Boston, MA, 2008; pp 1980–1987, 10.1007/978-0-387-48998-8_1532.

[43] Kristiansen K. A., Dalheim M. Ø., Christensen B. E. (2013). Periodate Oxidation and Macromolecular
Compaction of Hyaluronan. Pure Appl. Chem..

[44] Dabbish E., Russo N., Sicilia E. (2020). Rationalization of the Superior Anticancer
Activity of Phenanthriplatin: An In-Depth Computational Exploration. Chem. - Eur. J..

[45] Czapar A. E., Zheng Y.-R., Riddell I. A., Shukla S., Awuah S. G., Lippard S. J., Steinmetz N. F. (2016). Tobacco
Mosaic Virus Delivery of
Phenanthriplatin for Cancer Therapy. ACS Nano.

[46] Teng B., Han Y., Zhang X., Xiao H., Yu C., Li H., Cheng Z., Jin D., Wong K.-L., Ma P., Lin J. (2018). Phenanthriplatin­(IV) Conjugated Multifunctional up-Converting Nanoparticles
for Drug Delivery and Biomedical Imaging. J.
Mater. Chem. B.

[47] Wilhelm S., Tavares A. J., Dai Q., Ohta S., Audet J., Dvorak H. F., Chan W. C. W. (2016). Analysis of Nanoparticle Delivery
to Tumours. Nat. Rev. Mater..

[48] Minervini T., Cardey B., Foley S., Ramseyer C., Enescu M. (2019). Fate of Cisplatin
and Its Main Hydrolysed Forms in the Presence of Thiolates: A Comprehensive
Computational and Experimental Study. Metallomics.

[49] Ohta S., Hiramoto S., Amano Y., Emoto S., Yamaguchi H., Ishigami H., Kitayama J., Ito T. (2017). Intraperitoneal Delivery
of Cisplatin via a Hyaluronan-Based Nanogel/in Situ Cross-Linkable
Hydrogel Hybrid System for Peritoneal Dissemination of Gastric Cancer. Mol. Pharmaceutics.

[50] Yao Y., Zhou Y., Liu L., Xu Y., Chen Q., Wang Y., Wu S., Deng Y., Zhang J., Shao A. (2020). Nanoparticle-Based Drug Delivery
in Cancer Therapy and Its Role in
Overcoming Drug Resistance. Front. Mol. Biosci..

[51] Yu B., Tai H. C., Xue W., Lee L. J., Lee R. J. (2010). Receptor-Targeted
Nanocarriers for Therapeutic Delivery to Cancer. Mol. Membr. Biol..

[52] De
Luca E., Wang Y., Baars I., De Castro F., Lolaico M., Migoni D., Ducani C., Benedetti M., Högberg B., Fanizzi F. P. (2023). Wireframe DNA Origami for the Cellular
Delivery of Platinum­(II)-Based Drugs. Int. J.
Mol. Sci..

[53] Chen H., Wang Y., Liu Y., Tang L., Mu Q., Yin X., Zheng L., Chen Y., Liu C. (2021). Delivery of Cationic
Platinum Prodrugs via Reduction Sensitive Polymer for Improved Chemotherapy. Small.

[54] Münster L., Capáková Z., Fišera M., Kuřitka I., Vícha J. (2019). Biocompatible Dialdehyde Cellulose/Poly­(Vinyl
Alcohol) Hydrogels with Tunable Properties. Carbohydr. Polym..

[55] Baribeau S., Chaudhry P., Parent S., Asselin É. (2014). Resveratrol
Inhibits Cisplatin-Induced Epithelial-to-Mesenchymal Transition in
Ovarian Cancer Cell Lines. PLoS One.

