# Supplementary material for: Thermoresponsive Hyaluronate-Based Nanogels for Enhanced Phenanthriplatin Delivery in Cisplatin-Resistant Ovarian Cancer
Source: Biomacromolecules. 2025 Jul 22;26(8):5232–44. doi: 10.1021/acs.biomac.5c00692 (PMC12344714; doi:10.1021/acs.biomac.5c00692)
Supplement: Supplementary file 1 [file bm5c00692_si_001.pdf]

Thermoresponsive hyaluronate-based nanogels for enhanced phenanthriplatin delivery in cisplatin-resistant ovarian cancer

Supplementary Information

Filip Latečka [1], Tamara Juriňáková [2], Lukáš Münster [1], Monika Muchová [1], Michal Masařík [2,3,4] Anton Kuchynski[3], Petr Humpolíček [1,5], Michaela Fojtů [2,3], Jan Vícha [1*]*

[1]Centre of Polymer Systems, Tomas Bata University in Zlín, tř. Tomáše Bati 5678, 760 01 Zlín, Czech Republic

[2]Department of Pathological Physiology, Faculty of Medicine, Masaryk University, Kamenice 5, CZ-625 00 Brno, Czech Republic

[3]Department of Physiology, Faculty of Medicine, Masaryk University, Kamenice 5, CZ-625 00 Brno, Czech Republic

[4]BIOCEV, First Faculty of Medicine, Charles University, Průmyslová 595, 252 50, Vestec, Czech Republic

[5]Department of Fat, Surfactant and Cosmetics Technology, Faculty of Technology, Tomas Bata University in Zlín, nám. T. G. Masaryka 5555, 760 01 Zlín, Czech Republic

Emails: michaelafojtu@gmail.com, jvicha@utb.cz

Table S1. The polydispersity index (PDI) of series A, B and C samples below and above *LCST* measured in UPW.

Code	*PDI* at 25 °C	*PDI* at 37 °C
01_DCH_A	0.80 ± 0.09	0.43 ± 0.03
01_DCH_B	0.51 ± 0.03	0.42 ± 0.05
01_DCH_C	0.44 ± 0.04	0.53 ± 0.02
02_DCH_A	0.87 ± 0.09	0.62 ± 0.08
02_DCH_B	0.66 ± 0.12	0.39 ± 0.03
02_DCH_C	0.45 ± 0.04	0.40 ± 0.06

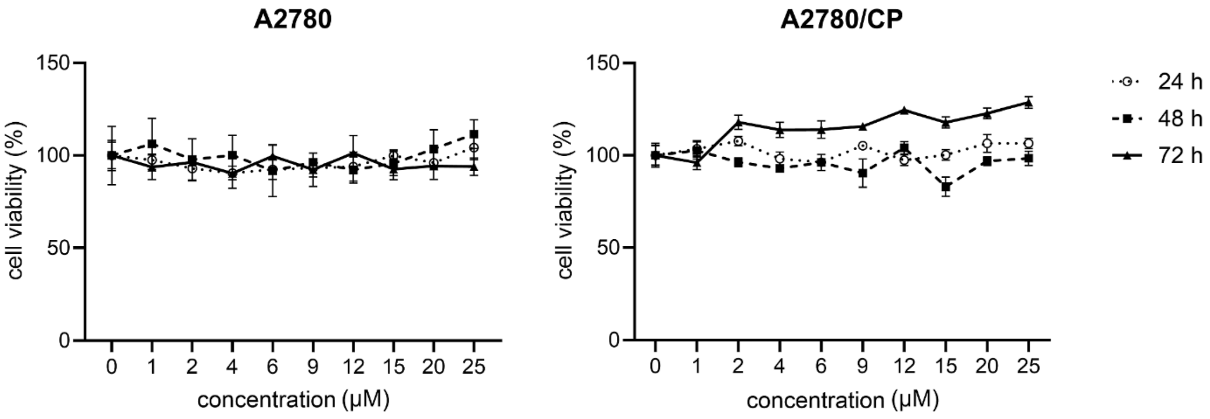

Figure S1. Cytotoxicity of 02_DCH_A evaluated in A2780 and A2780/CP cell lines across a concentration range of 0–25 µM at 24, 48, and 72 hours. Cell viability was assessed in tetraplicates, the results are displayed as mean ± SD.
